# Motherhood Imprints Tissue-Resident CD8^+^ Immunity for Long-Term Tissue Surveillance

**DOI:** 10.34133/research.1249

**Published:** 2026-04-10

**Authors:** Hui Zhang, Xiaojun Cai, Quazi T. H. Shubhra

**Affiliations:** ^1^ College of Veterinary Medicine, South China Agricultural University, Guangzhou 510642, China.; ^2^ School and Hospital of Stomatology, Wenzhou Medical University, Wenzhou 325027, China.; ^3^ Institute of Chemistry, University of Silesia in Katowice, 40-006 Katowice, Poland.

## Abstract

A full reproductive cycle (pregnancy followed by lactation and involution) imprints long-lived CD8^+^ tissue-resident-memory-like T cells in the breast. These cells restrain tumor growth in mice and are linked to a more inflamed tumor microenvironment and better outcomes in human triple-negative breast cancer.

Breast cancer remains the most frequently diagnosed malignancy among women worldwide and a leading cause of cancer-related mortality [1,2]. Despite major advances in screening and systemic therapies, disease progression and recurrence remain substantial clinical challenges, particularly in aggressive subtypes such as triple-negative breast cancer (TNBC). Increasing evidence indicates that tumor evolution is profoundly shaped by interactions with the tumor microenvironment, where malignant cells deploy diverse immune-evasion strategies to undermine effective antitumor surveillance [3,4]. Epidemiological studies indicate that parity reduces lifetime breast cancer risk (particularly for hormone-receptor-positive malignancies), whereas prolonged breastfeeding offers more specific protection against TNBC [5]. Classical models attributed this parity-induced protection to the terminal differentiation of mammary epithelial cells during pregnancy, which limits proliferative potential and susceptibility to oncogenic transformation [5]. Yet, this epithelial-centric view falls short of explaining why protection can persist for decades and why it is particularly evident in TNBC. Rather, the reproductive cycle represents an immunologically and stromally active phase in which the mammary epithelium, its surrounding stroma, and the associated vasculature undergo extensive remodeling (driven by coordinated epithelial–stromal–immune interactions) [4]. These transitions constitute one of the most dynamic inflammatory and regenerative events in adult tissues, suggesting that reproductive history may durably reprogram the mammary immune microenvironment. In this context, the recent study by Virassamy et al. [6] provides critical mechanistic insight into how the reproductive cycle imprints long-term immune surveillance in mammary tissue.

Against this backdrop, several unanswered questions persisted: (a) the lack of a long-lived, causal cellular mechanism linking reproductive history to tumor restraint; (b) uncertainty about which immune subsets persist after lactation and how they are induced and maintained; and (c) limited evidence that any such program is functionally protective rather than correlative. Prior studies have implicated immune processes, including dendritic-cell-mediated T-cell priming and chemokine-guided immune recruitment, and have underscored the prognostic importance of CD8^+^ tumor-infiltrating lymphocytes in TNBC [7]. However, they did not establish that a full reproductive cycle installs a resident, self-maintaining CD8^+^ antitumor compartment within the intraepithelial and periductal zones of the breast.

To resolve these uncertainties, Virassamy and colleagues [6] evaluated a tissue-resident memory T cell (T_RM_)-centric mechanism. The rationale is that T_RM_ biology suits prevention at an epithelial barrier: locality (nonrecirculating sentinels near ducts), longevity (niche self-renewal), and speed (rapid recall). They pair human single-cell and bulk profiling with spatial and flow-cytometric readouts and then use mouse models to test cause and effect. This work reframes parity-linked protection as an immunological imprint, a physiological program that educates and sustains resident CD8^+^ surveillance in the mammary gland (Fig. 1).

**Fig. 1:**
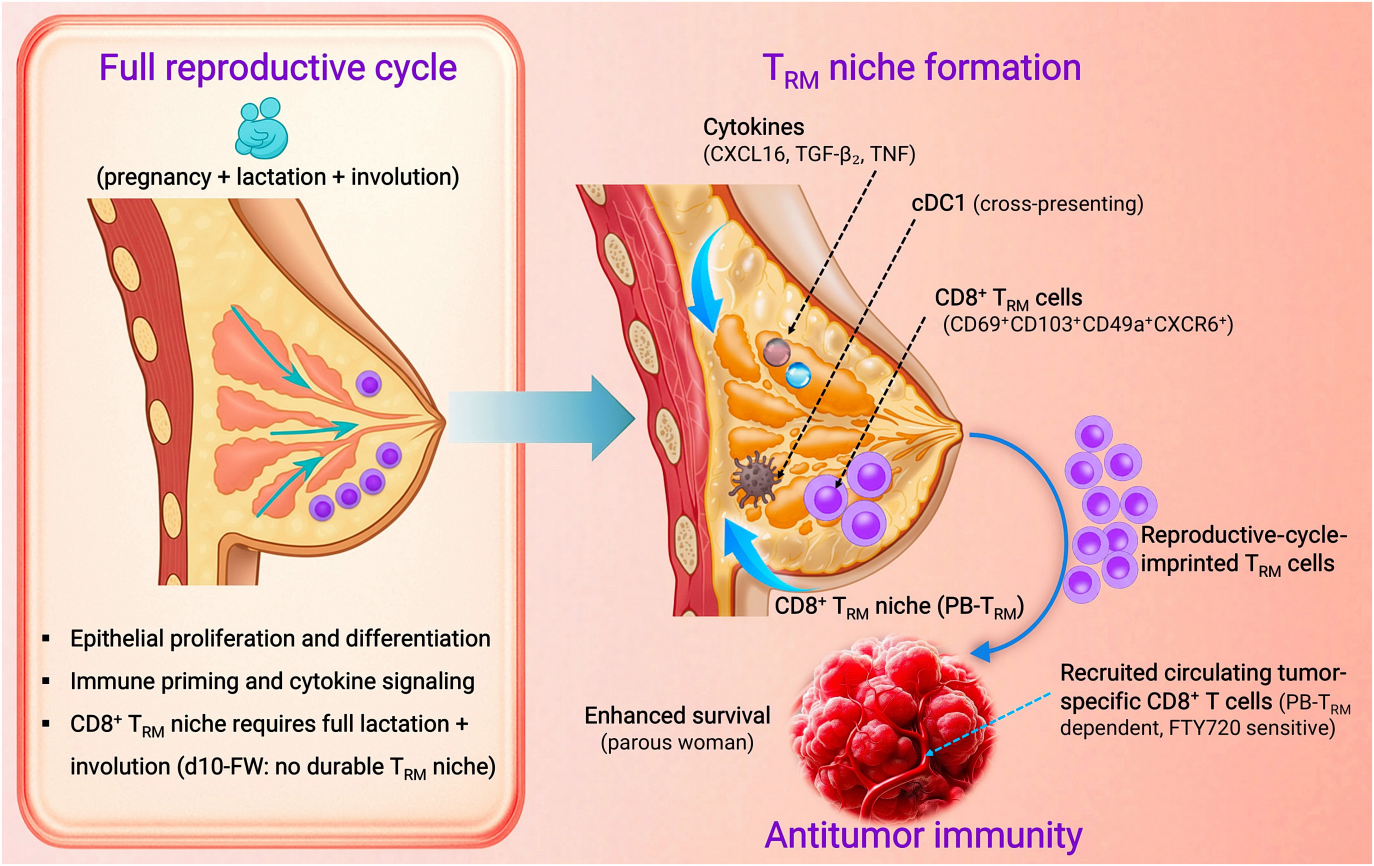
Full reproductive-cycle-associated CD8^+^ tissue-resident-memory-like T cell (T_RM_-like) niche and cooperative antitumor immunity. Conceptual schematic summarizing the key findings reported by Virassamy et al. [6]. The mammary gland is illustrated using a simplified human breast schematic for conceptual visualization. Pregnancy, sustained lactation, and physiological involution are associated with the enrichment of a parous breast tissue-resident memory CD8^+^ T cell population (PB-T_RM_) in the mammary gland, alongside T_RM_-associated cues (CXCL16, TGF-β2, and TNF) and cross-presenting conventional type 1 dendritic cells (cDC1). Mechanistic evidence from mouse models demonstrates that completion of lactation followed by involution establishes this durable CD8^+^ T_RM_ niche. Upon tumor challenge in mouse models, resident T_RM_-like cells cooperate with recruited circulating tumor-reactive CD8^+^ T cells (FTY720 sensitive) to enhance control of triple-negative breast cancer. d10-FW denotes day 10 post early force-weaning in the mouse model, a condition used to evaluate the requirement of sustained lactation for T_RM_ niche establishment. Some components of this figure were created with BioRender.com.

A core strength of this work is how elegantly the methodological choices serve the central question. Across 4 human datasets, parous tissue contains higher proportions of CD3^+^, CD8^+^, and T_RM_ CD8^+^ cells. They then confirm these findings with independent methods that add clear cell counts and spatial context. In cancer-free prophylactic mastectomy samples from women, flow cytometry shows increased CD69^+^CD103^+^ T_RM_ CD8^+^ cells, and spatial imaging places them within intraepithelial and periductal regions. Because these tissues are cancer-unaffected, the T_RM_-like enrichment is observed in nonmalignant human breast tissue, consistent with a preexisting immune state. To define this state molecularly, bulk RNA sequencing of human breast T_RM_ CD8^+^ cells versus matched circulating CD8^+^ cells identifies a residency-linked program with increased ITGAE, ITGA1, and CXCR6, which the authors term the parous breast T_RM_ (PB-T_RM_) signature. Together, these human tissue analyses support a long-lived, tissue-anchored CD8^+^ T_RM_ state in normal breast that is associated with parity. This human tissue foundation is important because it frames parity as a lasting shift in local immune architecture, rather than a transient hormonal exposure.

Building on this baseline, the authors connect PB-T_RM_ biology to human tumors and then test causality in mouse models. The PB-T_RM_ signature holds across independent patient cohorts and aligns with more immune-inflamed tumors in women with parity and even more so with breastfeeding. Clinically, breastfeeding has been reported to be associated with improved overall survival in a high-risk hormone-receptor-negative patient cohort [6]. However, as this signal comes from observational analyses, it must be interpreted as an association rather than causation and may be influenced by residual confounding. Mechanistic experiments in mice then clarify when the niche is established and identify key requirements for protection. In these models, completed lactation followed by full postlactational involution expands the mammary CD8^+^/T_RM_ compartment and limits later tumor growth, whereas early weaning does not. The protection in mice depends on CD8^+^ T cells, since depleting CD8α or CD8β removes the benefit [6]. Blocking lymphocyte egress in the mouse model with FTY720 also abolishes tumor control and suggests that resident T_RM_ work with recruited T cells during tumor challenge [8]. Together, the results support a clear mechanistic arc: lactation followed by full involution installs and maintains a CD8^+^ T_RM_-enriched niche that is ready to respond when malignancy emerges [6].

This work helps bridge an important gap by linking parity and sustained lactation to a durable, CD8^+^ T_RM_-enriched immune niche in the breast. First, the antigen specificity of the resident CD8^+^ pool remains undefined: whether these cells are maintained by self-antigens, microbe-related cues, or recurrent tumor-associated targets is not yet known. Clarifying this will determine whether these cells can be safely boosted or redirected by vaccines, microbiome cues, or tumor-directed immunotherapies. Second, the reported durability of human T_RM_-like enrichment is inferred from cross-sectional tissue spanning decades. Prospective, longitudinal follow-up could clarify the longevity and function of this memory state. Such data are essential to know whether this imprint is stable, remodeled by aging and therapy, or amenable to late-life intervention. Third (critical for broad applicability), the observed clinical signal is strongest in basal-like/TNBC. Its relevance in hormone-receptor-positive disease remains to be defined.

To translate these insights, future studies should prospectively test the clinical utility of the PB-T_RM_ niche. This means enrolling cohorts with carefully recorded reproductive histories and evaluating whether the PB-T_RM_ transcriptomic signature or the spatial density of CD69^+^CD103^+^CD8^+^ T cells near ducts is associated with prognosis or therapeutic outcomes. Because longitudinal sampling of normal human breast tissue is limited to occasional biopsies or surgical specimens, direct assessment of T_RM_ persistence remains challenging. Imaging or blood-based sampling could be explored as surrogate readouts, as is already being investigated for other immunological biomarkers [9]. Preclinical studies should also define the optimal postweaning interval during which the T_RM_-supportive niche is established and stabilized.

Looking ahead, 2 high-priority translational strategies follow naturally. One is biomarker-driven risk adaptation. It then applies the PB-T_RM_ transcriptomic signature and the spatial density of CD69^+^CD103^+^CD8^+^ T cells near ducts to refine patient stratification, particularly where TNBC risk and a T-cell-inflamed microenvironment are suspected. Other cancers already use immune signatures to guide prognosis and treatment, providing a model that breast cancer management could similarly follow [10].

The other is local niche engineering. Rational engineering of tissue immune niches is increasingly recognized as a way to boost local antitumor immunity while minimizing systemic toxicity [11]. To make this practical, the next step is to define the minimal T_RM_-supporting cues and deliver them locally, and then test feasibility by sustained periductal CD8^+^ T_RM_-like enrichment with minimal systemic immune perturbation. In rigorously controlled preclinical models, targeted delivery of the relevant cues (e.g., transforming growth factor beta 2 and tumor necrosis factor) together with conventional type 1 dendritic cells support could test whether the postinvolution, T_RM_-supportive niche can be safely recreated. Advanced drug-delivery platforms (particularly those designed to target the tumor microenvironment [12]) may enable such immunomodulatory cues to be spatially confined within defined tissue niches, including the mammary microenvironment, thereby enabling the localized reconstruction of T_RM_-supportive signaling circuits while minimizing systemic immune perturbation. Importantly, such approaches would likely be most relevant in defined clinical contexts, such as individuals at elevated risk of TNBC or patients undergoing treatment for breast cancer, rather than as universal preventive interventions. These findings do not suggest pregnancy or lactation as prevention; rather, they could inform targeted, localized interventions. Any future application should aim to emulate the resolved postinvolution state rather than the transient postpartum inflammatory phase, with appropriate safety monitoring.

In conclusion, the study by Virassamy et al. [6] outlines a physiological blueprint for durable, localized immunoprevention. The next steps are to define antigen specificity, confirm long-term persistence, and test niche-stabilizing interventions, aiming to translate this natural immune imprint into a clinically actionable tool.

## References

[1] Siegel RL, Kratzer TB, Giaquinto AN, Sung H, Jemal A. Cancer statistics, 2025. CA Cancer J Clin. 2025;75(1):10–45. 39817679 10.3322/caac.21871 PMC11745215

[2] Lin A, Ye P, Li Z, Jiang A, Liu Z, Cheng Q, Zhang J, Luo P. Natural killer cell immune checkpoints and their therapeutic targeting in cancer treatment. Research. 2025;8:0723. 40463500 10.34133/research.0723 PMC12131497

[3] Zhang Y, Feng G, He T, Yang M, Lin J, Huang P. Traceable lactate-fueled self-acting photodynamic therapy against triple-negative breast cancer. Research. 2024;7:0277. 40771576 10.34133/research.0277 PMC12326368

[4] Baxevanis CN, Fortis SP, Perez SA. The balance between breast cancer and the immune system: Challenges for prognosis and clinical benefit from immunotherapies. Semin Cancer Biol. 2021;72:76–89. 31881337 10.1016/j.semcancer.2019.12.018

[5] Russo J, Moral R, Balogh GA, Mailo D, Russo IH. The protective role of pregnancy in breast cancer. Breast Cancer Res. 2005;7(3):131–142. 15987443 10.1186/bcr1029 PMC1143568

[6] Virassamy B, Caramia F, Savas P, Harris MA, Pan J-W, Wang J, Brown E, O’Malley MMR, van Geelen CT, Hun M, et al. Parity and lactation induce T-cell-mediated breast cancer protection. Nature. 2026;649(8096):449–459. 41115453 10.1038/s41586-025-09713-5 PMC12779547

[7] Sebastião AI, Simões G, Oliveira F, Mateus D, Falcão A, Carrascal MA, Gomes C, Neves B, Cruz MT. Dendritic cells in triple-negative breast cancer: From pathophysiology to therapeutic applications. Cancer Treat Rev. 2025;133: Article 102884. 39837068 10.1016/j.ctrv.2025.102884

[8] Mao X, Tang X, Pan H, Yu M, Ji S, Qiu W, Che N, Zhang K, Huang Z, Jiang Y, et al. B cells and IL-21-producing follicular helper T cells cooperate to determine the dynamic alterations of premetastatic tumor draining lymph nodes of breast cancer. Research. 2024;7:0346. 38559676 10.34133/research.0346 PMC10981934

[9] Salawu A, Hernando-Calvo A, Chen RY, Araujo DV, Oliva M, Liu ZA, Siu LL. Impact of pharmacodynamic biomarkers in immuno-oncology phase 1 clinical trials. Eur J Cancer. 2022;173:167–177. 35872510 10.1016/j.ejca.2022.06.045

[10] Valenza C, Trapani D, Fusco N, Wang X, Cristofanilli M, Ueno NT, Curigliano G. The immunogram of inflammatory breast cancer. Cancer Treat Rev. 2023;119: Article 102598. 37437342 10.1016/j.ctrv.2023.102598

[11] Morris AH, Orbach SM, Bushnell GG, Oakes RS, Jeruss JS, Shea LD. Engineered niches to analyze mechanisms of metastasis and guide precision medicine. Cancer Res. 2020;80(18):3786–3794. 32409307 10.1158/0008-5472.CAN-20-0079 PMC7501202

[12] Pan H, Yang S, Gao L, Zhou J, Cheng W, Chen G, Shuhang W, Li N, Veranič P, Musiol R, et al. At the crossroad of nanotechnology and cancer cell membrane coating: Expanding horizons with engineered nanoplatforms for advanced cancer therapy harnessing homologous tumor targeting. Coord Chem Rev. 2024;506: Article 215712.

